# Facilitators and Barriers of Adherence to Time-Restricted Eating in Individuals with Type 2 Diabetes: A Qualitative Study

**DOI:** 10.3390/nu18091467

**Published:** 2026-05-04

**Authors:** Brooke L. Devlin, Siobhan McKenna, Bridget E. Radford, Rebecca C. Hall, Leah Brennan, Xochitl de la Piedad Garcia, Evelyn B. Parr

**Affiliations:** 1 School of Human Movement and Nutrition Sciences, The University of Queensland, Brisbane, QLD 4072, Australia; 2 Centre for Human Performance and Metabolism, Mary MacKillop Institute for Health Research, Australian Catholic University, Melbourne, VIC 3065, Australiabridget.radford@health.vic.gov.au (B.E.R.); evelyn.parr@acu.edu.au (E.B.P.); 3 School of Psychology and Public Health, La Trobe University, Wodonga, VIC 3690, Australia; leah.brennan@latrobe.edu.au; 4 School of Behavioural and Health Sciences, Australian Catholic University, Melbourne, VIC 3065, Australia; xochitl.delapiedadgarcia@acu.edu.au

**Keywords:** dietary adherence, nutrition support, lifestyle intervention, qualitative research, glycaemic control, behaviour change, dietary patterns, primary care, diabetes education

## Abstract

Background/Objectives: Time-restricted eating (TRE) has shown promise for improving glycemic control and supporting weight management in individuals with Type 2 Diabetes Mellitus (T2DM). However, limited evidence exists regarding factors that influence adherence to TRE in this population. This study aimed to explore the experiences of adults with T2DM who completed a 6-month TRE intervention to identify key barriers and enablers in adherence. Methods: A qualitative design was used involving semi-structured interviews with twenty-two adults (12 male, 10 female) with T2DM who completed a 6-month TRE intervention involving a 9 h eating window ending at 7:00 pm. Interviews were transcribed verbatim and analyzed inductively using thematic analysis. Results: Facilitators included (1) social and professional support; (2) simplicity; (3) perceived physical benefits such as weight loss, improved glycemic control, and sleep; and (4) improved routine. Barriers included (1) social events occurring outside the eating window; (2) misalignment of eating times with daily routines; and (3) hunger outside permitted eating hours. Although hunger was common, it did not consistently undermine adherence. Conclusions: TRE was viewed as acceptable and feasible for adults with T2DM when supported through structured implementation. Future interventions should incorporate social support and tailor strategies to individual routines to enhance sustainability.

## 1. Introduction

The global prevalence of Type 2 Diabetes Mellitus (T2DM) is rising, posing significant challenges to the individual and healthcare system due to its chronic nature and complex pathophysiology [1]. Without appropriate management, T2DM can lead to serious complications, including cardiovascular disease, stroke, kidney failure, and neuropathy, highlighting the need for effective and sustainable interventions [2,3,4]. The need is further amplified by the close interrelationship between T2DM, obesity and mental health, underscoring the importance of management strategies that extend beyond glycemic control to address body weight and psychological well-being [2,5].

Dietary change and physical activity are recognized as first-line treatments for managing T2DM, either alongside or prior to pharmacological intervention. Clinical guidance typically emphasizes improved diet quality, reduced consumption of discretionary foods, portion control, energy restriction for weight management, increased physical activity and reduced sedentary behaviour [6]. Diet and exercise interventions have demonstrated improvements in glycemic control and reduced body weight [7]. Notably, weight loss of approximately 15 kg has been shown to induce remission of T2DM in ~80% of cases [8], while 7% reduction in body weight is associated with significant improvements in insulin sensitivity [9]. Despite these benefits, long-term adherence to dietary interventions remains challenging, with weight regain common, and sustained adherence is the strongest predictor of success [10,11,12].

Adherence to dietary recommendations is particularly difficult for individuals with T2DM due to the complex demands of diabetes self-management, which require balancing medication use, physical activity, and dietary changes. These challenges are compounded by the obesogenic environment, characterized by easy access to energy-dense foods and sedentary lifestyles [13]. Moreover, food plays a central social and cultural role, creating further barriers to sustained dietary change [14,15]. Low adherence to T2DM dietary interventions is well documented [16,17], prompting recent recommendations from American Diabetes Association for pragmatic, routine-based approaches to healthy eating [18]. Similarly, recent Australian policy reviews highlight the urgent need for novel and scalable dietary strategies [19].

Emerging research into time-restricted eating (TRE) offers an alternative dietary approach that aligns food intake with circadian rhythms, potentially improving metabolic function through synchronization of peripheral clocks [20,21,22,23]. By restricting energy intake to a defined daily eating window, TRE may support weight loss and improved glucose metabolism [21,22,23,24,25]. Initial trials of TRE have demonstrated favourable metabolic outcomes [26,27], and the simplicity of TRE may enhance feasibility and acceptability [28,29]. Qualitative studies in populations with overweight, obesity, and those at high diabetes risk consistently identify facilitators such as simplicity and improved routine, alongside barriers including social pressures, work schedules and hunger [28]. Despite this expanding literature, qualitative work specifically examining the experiences of individuals with T2DM, a population managing complex treatment demands and heightened risk of diabetes-related distress, remains limited.

This qualitative study aims to explore the barriers and facilitators to adherence to TRE in individuals with T2DM, providing insights to inform future dietary interventions and support translation into clinical practice.

## 2. Materials and Methods

### 2.1. Study Design

A cross-sectional, qualitative study design explored barriers and facilitators in adherence to TRE in participants with T2DM. This study adopted a qualitative, inductive approach underpinned by a constructivist paradigm to gain more nuanced insights on TRE and its applicability in the real world. This work forms part of a larger trial investigating the effect of TRE compared to diet modification in people with T2DM (ANZCTR Registration: ACTRN12620000453987, approval date: 8 April 2020) [21]. The study received ethical approval from the participating university (Australian Catholic University Human Ethics Committee: 2019-359HC) and was co-registered with The University of Queensland Ethics Committee (2022/HE001636). The interviews took place face-to-face at Australian Catholic University, Melbourne, during 2021 to 2022. All participants signed written consent. This study is reported according to the Consolidated Criteria for Reporting Qualitative Research.

### 2.2. Participants and Recruitment

As previously detailed [21], individuals met the following inclusion criteria: (a) aged 35 to 65 years, diagnosed with T2DM and baseline HbA1c of 6.5–≤10%, (b) taking no more than two oral hypoglycaemic agents (with the exception of sulphonylureas) and no use of insulin or GLP-1 agonists, (c) body mass index (BMI) of 25–45 kg/m^2^. Participants were screened for eating disorder risk using the EAT26, a 26-item self-report measure, and were ineligible to participate if they scored ≥20 (i.e., presented disordered eating patterns) [30]. Other exclusions included a baseline eating window of <12 h (self-reported), previous bariatric surgery, a history of psychotic disorders, a current diagnosis of other major psychiatric illness, or a change in medication within 3 months. Participants were recruited through National Diabetes Service Scheme (NDSS) emails, social media advertisements, flyers, and databases from previous studies.

The intervention duration was 6 months, with participants being required to follow either a time-restricted eating protocol (TRE) or individually prescribed dietary advice from an Accredited Practising Dietitian (DIET), based on random allocation. Participants were allocated to the intervention group stratified by sex (male/female) and baseline HbA1c (i.e., ≥6.5–≤8%, >8–≤10%) using block randomisation. The TRE group were required to limit their energy consumption to within 10 am to 7 pm for as many days as possible during the 6-month research period. No dietary education was provided to the TRE group. Participants in both groups attended consultations with the research dietitian (1 × 1 h, 3 × 30 min) to provide education, encourage compliance and discuss adherence, barriers and strategies relative to the allocated group. Upon completion of the intervention, all participants in the TRE group (*n* = 22) were invited to participate in the qualitative interview. This study focuses on the qualitative exploration of barriers and facilitators in the TRE group, drawn from interviews conducted at the end of the 6-month intervention.

### 2.3. Data Collection

The semi-structured interviews were conducted by a member of the research team (EBP, BER, SM). A semi-structured interview guide was developed based on the literature and prior research conducted by the research team and included questions to explore barriers and facilitators alongside rating scales to understand the participants’ study experience [29] (Table 1). Interviews were conducted in person (with only the interviewer and participant present) with written notes taken during the interview. All interviews were audio-recorded and transcribed verbatim for analysis. Interviews lasted approximately 15 to 20 min.

### 2.4. Data Analysis

Transcripts were imported into NVivo (NVivo qualitative data analysis software, Version 12, QSR International 14 Pty Ltd., Melbourne, VIC, Australia). Thematic analysis was guided by the Consolidated Criteria for Reporting Qualitative Research (COREQ) [31]. Data analysis was exploratory and inductive and followed phases of reflexive thematic analysis; familiarizing with the data, generating initial codes, searching for themes, reviewing themes and defining themes [32,33]. Two members of the research team (BLD, SM) reviewed and refined primary themes. Direct quotes from transcripts were included to demonstrate researchers’ interpretation of raw data. Agreement was reached on four overarching themes and chosen quotations were reflective of the data.

## 3. Results

Interview transcripts from all 22 participants (12 males and 10 females) who completed the TRE intervention were analyzed to identify barriers and facilitators to adherence. Participant characteristics are presented in Table 2.

Thematic analysis of the interviews identified four facilitating themes (Table 3) and three barrier themes (Table 4).

Support networks represented a key facilitator, encompassing family support and researcher/dietitian support. Family members facilitated adherence by participating in TRE or adjusting shared mealtimes. Researcher and dietitian support promoted adherence through practical strategies, assistance with managing social situations, and accountability via regular check ins which helped participants remain or get ‘back on track’.

Ease of implementation was a facilitator, with participants describing TRE as simpler and more desirable than previous diets. Limiting eating to a defined time window was perceived as less burdensome than restricting food groups or monitoring dietary intake. Participants valued the reduced cognitive load associated with TRE, as it eliminated calorie counting and detailed meal planning, making the approach feel more sustainable.

Observed physical change was the third facilitating theme. Weight loss, improved blood glucose, and better sleep quality motivated continued adherence. Improvements in sleep were attributed to a more consistent routine, earlier evening meals, and sufficient time between dinner and bedtime.

Improved routine was the fourth facilitating theme, with participants reporting greater evening structure, increased time for family or leisure, and enhanced daily organization after shifting dinner earlier.

Social commitments represented a major barrier to adherence, with participants reporting that a 7 pm eating window restricted social engagements and created pressure to eat in social contexts. Routine misalignment also posed challenges; work schedules, late finishes, or evening exercise often conflicted with the prescribed eating window. Hunger outside the eating window was the final barrier, with some participants reporting persistent hunger led to non-adherence, although experiences varied regarding whether hunger diminished over time.

## 4. Discussion

This qualitative study explored the barriers and facilitators in adherence to a 9 h TRE protocol among individuals with T2DM over six months. Participants highlighted support networks, simplicity, perceived health benefits and improved routine as key facilitators, whereas social commitments, routine misalignment, and hunger impeded adherence. By exploring the perspectives of individuals navigating the challenges of T2DM, this study uniquely contributes to the literature by identifying barriers and facilitators to adhering to TRE. Understanding these perspectives is essential, as approximately 36% of individuals with T2DM experience diabetes-related distress, [34], which can negatively influence adherence to dietary recommendations. Our findings suggest that TRE, when applied in a pragmatic and supported manner, is acceptable as a dietary strategy. These insights offer valuable guidance for tailoring TRE interventions in practice to the needs of people living with T2DM, a population that is often underrepresented in behavioural nutrition research.

Consistent with prior studies in both TRE and other dietary interventions, social support significantly contributed to adherence to TRE, especially when family members adjusted mealtimes to align with TRE protocols [28,35,36,37]. The ease of implementation is a consistent theme throughout the qualitative TRE literature and other dietary interventions, with a regular compliment of the TRE strategy being its simplicity and not utilizing intentional energy restriction as a dietary method [35,36,38]. Diet interventions are often complicated, with both food selection and energy restrictions requiring significant planning into daily food consumption. This results in the need for significant and ongoing cognitive control that may be difficult to sustain, particularly during stressful times. Participants within this study reported that the freedom around what to eat provided by TRE facilitated their continued adherence to the protocol, with reduced monitoring and decision-making. The primary investigation to which this qualitative study is linked found higher adherence with TRE and higher adherence over time compared to standard dietetic practice [21]. These findings of increased adherence align with research on older adults, where adherence improved when TRE protocols allowed flexibility, such as a self-selected eating window and additional of an unrestricted eating day each week [39]. Minor adjustments to TRE protocols are consistent with recommendations for increasing adherence to TRE interventions, with studies that focus on self-selected eating windows reporting higher rates of adherence and lower participant drop out [20,37,38,40].

The TRE structure provided consistency, fostering behavioural changes such as earlier dinners, which enhanced evening family time. Routine-based adherence reflects findings across the TRE literature, where participants who establish structured eating patterns achieve greater long-term success [28,37]. It is to be noted that this prescribed 10 am to 7 pm time frame was identified as a barrier for some in this study, with reports that the protocol translated poorly into daily life and current daily routines. For example, some individuals who worked and were unable to eat exactly at or around 10 am had to further delay their first meal. Negative feelings around TRE due to incompatible work schedules are important in TRE adherence. Previous studies reported similar findings regarding complications with work schedules resulting in altered patterns in hunger and eating outside of the TRE window [28,36,41]. However, in a previous study, O’Connor et al. [36] utilized self-selected TRE windows to allow autonomy, increased motivation and adherence. Allowing participants to self-select their TRE window or adjust on days with different social or work commitments may be a key strategy to address this barrier, ensuring the eating window aligns with their daily life, work schedule, family commitments and social environment and thus facilitating greater adherence.

Importantly, evidence suggests that self-selected windows do not negate the proposed mechanisms of TRE. The primary metabolic benefits, such as improved insulin sensitivity, reduced postprandial glucose excursions, and alignment of peripheral clocks, are driven by restricting food intake to a consistent, consolidated period rather than the exact timing of that window. Therefore, flexibility in start and end times can maintain circadian alignment and metabolic advantages, provided the eating window remains within the active phase of the day and avoids late-night eating, which is associated with impaired glucose regulation. Studies indicate the early TRE (eTRE) may confer additional benefits for glycemic control compared to later windows, but self-selected windows still improve metabolic outcomes when they avoid late-night eating [42].

Physical changes and perceived health benefits such as weight loss, improved glucose levels, improved quality of life and better sleep motivated continued adherence, corroborating similar research exploring facilitators to TRE adherence [39,40,41,43,44]. It is important to note that a lack of change or negative changes in these measures were not identified as barriers to adherence. Other studies that have found physical change to be a driver of TRE adherence have also identified it as a barrier, with a lack of desired weight loss and negative feelings around health (e.g., tiredness) influencing adherence [36]. A potential explanation for this is that weight loss and sleep were not a focus in the current study and therefore participants were not expecting improvements in this area and thus the lack of changes was not perceived to be a barrier while positive changes were perceived as a bonus. While the emerging literature continues to explore the potential cardiometabolic implications of TRE, the present study was not designed to assess health outcomes and instead focused on behavioural factors influencing adherence.

The main barriers in adhering to TRE included social environments, routine incompatibility and hunger. Social environments, particularly eating outside the TRE window during social events, were challenging. It is well established that social norms play a significant role in dietary behaviour [45]. As such, social environments can act as both facilitators and barriers to successful completion of a diet intervention, with support from family and friends and an absence of social pressures driving adherence. Most participants expressed that a lack of adherence to TRE during a social event was a necessity, with eating and drinking being an important part of socialization. These findings are consistent with the previous literature that, similarly, reported that participants broke the protocol for social outings [28]. To manage this barrier, future interventions could incorporate flexible strategies such as allowing occasional “social exception” days, thereby supporting adherence without compromising social engagement.

Feelings of hunger represented a common barrier and have been shown to decrease over the course of a TRE intervention in prior research [35]. This could be a result of participants adjusting to the intervention and reinforcing their natural circadian rhythm. Evening and bedtime hunger were more commonly reported within this study, with reports of morning hunger lessening during the study or being easier to manage. These findings are supported within the literature, with a systematic review of TRE and appetite in adults concluding that bedtime appetite is increased by TRE, whilst morning appetite is decreased or remains unchanged [46]. With hunger varying between individuals, accounting for when feelings of hunger are experienced as well as the level of discomfort it causes will determine how significant a barrier hunger presents.

A key strength of this study is the in-depth qualitative exploration of the barriers and facilitators to TRE adherence among individuals with T2DM, providing valuable insights to inform future research and support effective implementation of TRE in real-world clinical and community settings. A total of 12 male participants took part in the interviews, which is a strength. Male representation in qualitative research on dietary interventions is typically low, as men are less likely to enrol in nutrition-related studies and even less likely to participate in qualitative components. Including a substantial number of male participants enhances the diversity of perspectives and improves the generalizability of findings across genders. However, the differences in barriers and facilitators between males and females were not compared. Further, it should be noted the study includes a total of 22 participants, which does limit representativeness. The age of the participants can be viewed as both a strength and limitation of the research. The average age of participants (53.4 ± 9.2 years) is reflective of a group of individuals with T2DM diagnosed within the last 7–10 years, where the age of onset of T2DM has been reported previously to be ~50.3 years [47]. A commonly reported barrier to TRE was the impracticality of the protocol and the influence on social lives. Considering this, the barriers reported could be expected to be different in younger participants, especially those with younger children and families. Another limitation of the study is that all participants were highly motivated and educated and contacted the research team themselves to join the study and may not represent individuals following TRE outside of a research context. Additionally, only participants who completed the intervention were included in this qualitative component.

From a primary care perspective, these findings support TRE as a pragmatic dietary option for motivated adults with T2DM when delivered flexibly. Codesigning an 8–10 h eating window that fits work, family commitments, and chronotype while avoiding late-night eating may improve feasibility and adherence. Light-touch support, including brief dietitian or diabetes educator check ins and practical strategies for managing social occasions, appears beneficial.

The findings from this study raise several important questions for future clinical and implementation research. One key area for investigation is the degree of flexibility that can be incorporated into TRE protocols without attenuating metabolic benefits. For example, it remains uncertain whether allowing a pre-planned weekly ‘social exception’ meal or day could improve adherence and quality of life while maintaining glycemic and metabolic outcomes. Additionally, questions remain regarding individual variability in response to early vs late TRE, and whether integrating chronotype and work schedules into decision-support tools could better balance circadian alignment with real-world feasibility, thereby improving long-term HbA1c and adherence. Finally, the observed relationships between sleep quality, hunger and eating time warrant further exploration, particularly whether earlier dinner enhances sleep efficiency.

## 5. Conclusions

TRE appears acceptable for individuals with T2DM, with adherence influenced by external support, ease of implementation, routine improvement and physical benefits. Findings emphasize importance of self-selected eating windows and flexibility to overcome barriers such as work schedules, social pressures and hunger for increased adherence. Future research should explore personalized TRE interventions to further maximize adherence and long-term efficacy in diverse T2DM populations.

## Figures and Tables

**Table 1:** Semi-structured interview guide exploring adherence to TRE dietary intervention in individuals with T2DM.

1. Based on your experience to date, what are your general thoughts and reflections on following TRE since starting the study?
2. What, if anything, have you enjoyed about the intervention period you have completed so far with respect to dietary intake and following advice?
3. What, if anything, have you not enjoyed about the intervention period you have completed so far, with respect to dietary intake and following advice?
4. Can you describe any barriers or challenges you have experienced when trying to adhere to TRE?
5. Can you describe any aspects of TRE that you have found appealing or helpful?
6. How have the dietary changes you have implemented so far made you feel physically, emotionally and psychologically?
7. Considering your home/work and broader life context, how would you describe your experience with adhering to TRE over the course of the study?
8. Considering your home/work and broader life context, can you comment on the researcher support throughout the study intervention?
9. How have the changes to your dietary intake or eating patterns influenced your hunger levels?
10. Have the changes to your dietary intake/patterns influenced your social engagements? If so, how?
11. Have the changes to your dietary intake/patterns influenced your lifestyle, i.e., daily patterns including sleep, activity and household duties? If so, how?
12. How does the advice you have been given compare to other “diets” you have tried?
13. What changes, if any, have you noticed to your overall health in the study period so far?
14. Is there anything else you would like to share about your experiences, thoughts or opinions regarding the dietary approach you were assigned?

**Table 2:** Participant characteristics.

	All (*n* = 22)	Males (*n* = 12)	Females (*n* = 10)
Age (y)	53.4 ± 9.2	49.2 ± 9.2	58.4 ± 6.5
Body mass (kg)	87.9 ± 13.0	90.9 ± 12.6	84.2 ± 13.2
Height (m)	1.68 ± 0.08	1.73 ± 0.04	1.61 ± 0.07
BMI (kg/m^2^)	31.1 ± 4.1	30.2 ± 4.2	32.2 ± 3.8
Years diagnosed with T2DM	7.0 ± 3.7	5.1 ± 2.7	9.2 ± 3.5

**Table 3:** Themes, subthemes and supporting quotations of facilitators regarding adherence to time-restricted eating in individuals with T2DM.

Theme	Subtheme	Supporting Quotations
Facilitators		
Support Networks	Family Support	*The family got on board so it made it easier. They were eating at the same time, so that made it easy. (58F)* *Everyone is supporting me which I’m really pleased and it helps (46M)*
	Dietitian and Research Team Support	*I’m improving, like it helped me a lot in every way with you guys (referring to research team), giving me the advice I needed and the support. (46M)* *The accountability, the more immediate feedback, you know if you’re coming in every week or two weeks getting back on task and focusing and doing measurements, so the accountability helped. So, if someone guiding you do more better than by doing by your own, so guidance is a lot important (48M)* *Well, the dietitian just gave a bit of extra help with what to do, some strategies (44F)*
Ease of Implementation		*I found it a lot easier, to adhere to TRE. It just takes away the decision part. Like any other diet you have to you have to prepare the food or take specific food, yeah, with this one you just have to look at the clock, and. Yeah, quite easy for me. To do what it’s asking for. (46M)* *Well, again, I didn’t have to change my diet. It was very it was a very simple regimen to follow. You don’t have to count your calories. You don’t have to you know, be mindful of the sugar content. Just eat between this time and that time and you’re all good… (63M)* *It was just very straightforward and very, very simple. (54M)* *I like this TRE because I can still eat what I want, I think I find it difficult if I am restricted to what I can eat. Yeah whereas the time is not such a big deal. (44F)* *I think it’s less difficult than other diets yeah ‘cause other diets you’re always counting calories and all sorts of things, You can’t eat this, you can’t eat that whereas this one you just have a window and that’s it. (43M)*
Physical Change	Weight Loss	*And physically the, the weight loss is a good positive sign, yes, weight loss is definitely a positive sign. So that emotionally also has motivated me more and obviously this happiness that you know I can feel. (44F)* *Within this time there has been I think improvement in my weight overall. (43M)* *I’m feeling better because people notice, are seeing, that I’m losing weight. (58F)*
	Improved Blood Glucose	*When I started it was 6.8 and now it’s reduced to 6.1. (54M)* *Yeah, I mean my sugar and diabetes has improved. (37M)*
	Sleep Quality	*I’d say I sleep better. It’s improved my sleep and I always feel more energy. Those two things are unmatched. (46M)* *It’s amazing how just changing the time of your dinner can really relax you and put you in a really deep sleep. (43F)*
Improved Routine and Habits	-	*Advice was simple, just take your dinner before 7. I used to have dinner after 10:00 PM you know so that kind of heavy dinner I mean with rice and all these things. Surely that is not that healthy. I mean I’d just straight go to the bed after. So this study broke that rule and habit, you know, so I just take my early dinner before 7, then I get a lot of time to digest and just do activities you know. (46M)* *So it discouraged, for example, me snacking, which is really good and I feel good about that. And I don’t eat out of a whim, like when I’m watching TV, I have totally taken that habit out, this is really good for me. (46F)*

**Table 4:** Themes and supporting quotations regarding barriers to adherence to time-restricted eating in individuals with T2DM.

Theme	Supporting Quotations
Social commitments after eating window	*The only issue is mainly around the social sometimes you know, in the night you most of the dinners, get togethers or going out with friends or family happens after 7:00 o’clock. It’s not like, you know, it’s a complete disappointment as such, but we could plan around it as well. But if I have to point out something. (49M)* *You know the social pressure to go to these sorts of evening things. They put a plate in front of me. If you are not eating, we waste these foods. So what do you do? (49M)*
Eating window not feasible with current routine	*So what I didn’t enjoy was probably because when we are working, I work full time and, I know I shouldn’t eat until 10:00 o’clock. But sometimes I can’t do it because I have a meeting at 10 that goes for two hours or an hour and I would be too hungry waiting until 11 or 12. And then you know you feel fatigued and tired so I need to eat before 10. I can’t keep it so strict everyday depending on my work. (37M)* *Because I’m an early bird it was a bit hard. I might get up at 5 in the mornings and for me to have breakfast at 10 it is really late, I’ve been up for 5 h already. (59F)* *The most difficult I found was in the morning as my situation is I have to go and start work at 9:30 or 10 but it’s the same time I have to have had breakfast. (49M)*
Hunger	*Yeah, but I had to eat ‘cos I’d get too hungry, hungry, hungry. (54M)* *Sometimes I start to feel I’m getting hungry and need to eat but I would test and sugars would be fine. I was just hungry. That was a challenge to get used to. (59F)*

## Data Availability

Data are not available publicly but are available on request from the corresponding author.

## References

[1] He K.-J., Wang H., Xu J., Gong G., Liu X., Guan H. (2024). Global burden of type 2 diabetes mellitus from 1990 to 2021, with projections of prevalence to 2044: A systematic analysis across SDI levels for the Global Burden of Disease Study 2021. Front. Endocrinol..

[2] Braunwald E. (2019). Diabetes, heart failure, and renal dysfunction: The vicious circles. Prog. Cardiovasc. Dis..

[3] Saeedi P., Petersohn I., Salpea P., Malanda B., Karuranga S., Unwin N., Colagiuric S., Guariguatad L., Motalae A.A., Ogurtsova K. (2019). Global and regional diabetes prevalence estimates for 2019 and projections for 2030 and 2045: Results from the International Diabetes Federation Diabetes Atlas, 9th edition. Diabetes Res. Clin. Pract..

[4] Khan M.A.B., Hashim M.J., King J.K., Govender R.D., Mustafa H., Al Kaabi J. (2020). Epidemiology of type 2 diabetes—Global burden of disease and forecasted trends. J. Epidemiol. Glob. Health.

[5] Ruze R., Liu T., Zou X., Song J., Chen Y., Xu R., Yin X., Xu Q. (2023). Obesity and type 2 diabetes mellitus: Connections in epidemiology, pathogenesis, and treatments. Front. Endocrinol..

[6] The Royal Australian College of General Practitioners (2020). Management of Type 2 Diabetes: A Handbook for General Practice.

[7] Marín-Peñalver J.J., Martín-Timón I., Sevillano-Collantes C., del Cañizo-Gómez F.J. (2016). Update on the treatment of type 2 diabetes mellitus. World J. Diabetes.

[8] Magkos F., Hjorth M.F., Astrup A. (2020). Diet and exercise in the prevention and treatment of type 2 diabetes mellitus. Nat. Rev. Endocrinol..

[9] Hays N.P., Starling R.D., Liu X., Sullivan D.H., Trappe T.A., Ehsani A.A., Evans W.J. (2004). Effects of an ad libitum low-fat, high-carbohydrate diet on body weight, body composition, and fat distribution in older men and women: A randomized controlled trial. Am. J. Clin. Nutr..

[10] Machado A.M., Guimarães N.S., Bocardi V.B., da Silva T.P.R., do Carmo A.S., Menezes M.C., Duarte C.K. (2022). Understanding weight regain after a nutritional weight loss intervention: Systematic review and meta-analysis. Clin. Nutr. ESPEN.

[11] Barte J.C.M., ter Bogt N.C.W., Bogers R.P., Teixeira P.J., Blissmer B., Mori T.A., Bemelmans W.J.E. (2010). Maintenance of weight loss after lifestyle interventions for overweight and obesity, a systematic review. Obes. Rev..

[12] Look AHEAD Research Group (2010). Long-term effects of a lifestyle intervention on weight and cardiovascular risk factors in individuals with type 2 diabetes mellitus: Four-year results of the Look AHEAD trial. JAMA Intern. Med..

[13] Westman E.C., Yancy W.S., Mavropoulos J.C., Marquart M., McDuffie J.R. (2008). The effect of a low-carbohydrate, ketogenic diet versus a low-glycemic index diet on glycemic control in type 2 diabetes mellitus. Nutr. Metab..

[14] Hill-Briggs F., Adler N.E., Berkowitz S.A., Chin M.H., Gary-Webb T.L., Navas-Acien A., Thornton P.L., Haire-Joshu D. (2021). Social determinants of health and diabetes: A scientific review. Diabetes Care.

[15] Wilson D., Diji A.K.-A., Marfo R., Amoh P., Duodu P.A., Akyirem S., Gyamfi D., Asare H., Armah J., Enyan N.I.E. (2024). Dietary adherence among persons with type 2 diabetes: A concurrent mixed methods study. PLoS ONE.

[16] Stephens M.A.P., Franks M.M., Rook K.S., Iida M., Hemphill R.C., Salem J.K. (2013). Spouses’ attempts to regulate day-to-day dietary adherence among patients with type 2 diabetes. Health Psychol..

[17] Trepanowski J.F., Kroeger C.M., Barnosky A., Klempel M.C., Bhutani S., Hoddy K.K., Gabel K., Freels S., Rigdon J., Rood J. (2017). Effect of alternate-day fasting on weight loss, weight maintenance, and cardioprotection among metabolically healthy obese adults: A randomized clinical trial. JAMA Intern. Med..

[18] American Diabetes Association (2022). Standards of medical care in diabetes—2022. Diabetes Care.

[19] House of Representatives Standing Committee on Health, Aged Care and Sport (2024). The State of Diabetes Mellitus in Australia.

[20] Manoogian E.N.C., Chow L.S., Taub P.R., Laferrère B., Panda S., Varady K.A. (2021). Time-restricted eating for the prevention and management of metabolic diseases. Endocr. Rev..

[21] Parr E.B., Devlin B.L., Radford B.E., Hawley J.A., Burke L.M. (2024). Comparing the effects of time-restricted eating on glycaemic control in people with type 2 diabetes with standard dietetic practice: A randomised controlled trial. Diabetes Res. Clin. Pract..

[22] Parr E.B., Steventon-Lorenzen N., Johnston R., Maniar N., Devlin B.L., Lim K.H.C., Hawley J.A. (2023). Time-restricted eating improves measures of daily glycaemic control in people with type 2 diabetes. Diabetes Res. Clinc Pract..

[23] Maqsood S., Amjad S., Ahmed F., Ahmad M.F. (2025). Time-restricted eating and circadian rhythms: A new frontier in diabetes and obesity management. Prim. Care Diabetes.

[24] Gill S., Panda S. (2016). A smartphone app reveals erratic diurnal eating patterns in humans that can be modulated for health benefits. Cell Metab..

[25] Uldal S., Clemmensen K.K.B., Persson F., Færch K., Quist J.S. (2022). Is time-restricted eating safe in the treatment of type 2 diabetes? A review of intervention studies. Nutrients.

[26] Nam T., Oh H., Kim A., Oh Y. (2025). Time-restricted eating improves glycemic control in patients with type 2 diabetes: A meta-analysis and systematic review. Int. J. Mol. Sci..

[27] Lin X., Guan Y., Wu G., Huang J., Wang S. (2022). Time-restricted eating for patients with diabetes and prediabetes: A systematic review. Front. Nutr..

[28] Bjerre N., Holm L., Veje N., Quist J.S., Færch K., Hempler N.F. (2022). What happens after a weight loss intervention? A qualitative study of drivers and challenges of maintaining time-restricted eating among people with overweight at high risk of type 2 diabetes. Appetite.

[29] Parr E.B., Devlin B.L., Lim K.H., Moresi L.N., Geils C., Brennan L., Hawley J.A. (2020). Time-restricted eating as a nutrition strategy for individuals with type 2 diabetes: A feasibility study. Nutrients.

[30] Garner D.M., Olmsted M.P., Bohr Y., Garfinkel P.E. (1982). The Eating Attitudes Test: Psychometric features and clinical correlates. Psychol. Med..

[31] Tong A., Sainsbury P., Craig J. (2007). Consolidated criteria for reporting qualitative research (COREQ): A 32-item checklist for interviews and focus groups. Int. J. Qual. Health Care.

[32] Braun V., Clarke V. (2006). Using thematic analysis in psychology. Qual. Res. Psychol..

[33] Braun V., Clarke V. (2019). Reflecting on reflexive thematic analysis. Qual. Res. Sport Exerc. Health.

[34] Perrin N.E., Davies M.J., Robertson N., Snoek F.J., Khunti K. (2017). The prevalence of diabetes-specific emotional distress in people with Type 2 diabetes: A systematic review and meta-analysis. Diabet. Med..

[35] Bjerre N., Holm L., Quist J.S., Færch K., Hempler N.F. (2021). Watching, keeping and squeezing time to lose weight: Implications of time-restricted eating in daily life. Appetite.

[36] O’Connor S.G., Boyd P., Bailey C.P., Nebeling L., Reedy J., Czajkowski S.M., Shams-White M.M. (2022). A qualitative exploration of facilitators and barriers of adherence to time-restricted eating. Appetite.

[37] Jan Y.C., Chiang S.W., Hsieh T.C. (2023). Exploring the successful experience of time-restricted eating in overweight adults: A qualitative study. Appetite.

[38] Bailey C.P., Boyd P., Shams-White M.M., Czajkowski S.M., Nebeling L., Reedy J., O’Connor S.G. (2024). Time-restricted eating in community-dwelling adults: Correlates of adherence and discontinuation in a cross-sectional online survey study. J. Acad. Nutr. Diet..

[39] Lee S.A., Sypniewski C., Bensadon B.A., McLaren C., Donahoo W.T., Sibille K.T., Anton S. (2020). Determinants of adherence in time-restricted feeding in older adults: Lessons from a pilot study. Nutrients.

[40] Wilkinson M.J., Manoogian E.N.C., Zadourian A., Lo H., Fakhouri S., Shoghi A., Wang X., Fleischer J.G., Navlakha S., Panda S. (2020). Ten-hour time-restricted eating reduces weight, blood pressure, and atherogenic lipids in patients with metabolic syndrome. Cell Metab..

[41] O’Neal M.A., Gutierrez N.R., Laing K.L., Manoogian E.N.C., Panda S. (2023). Barriers to adherence in time-restricted eating clinical trials: An early preliminary review. Front. Nutr..

[42] Liu J., Yi P., Liu F. (2023). The Effect of Early Time-Restricted Eating vs Later Time-Restricted Eating on Weight Loss and Metabolic Health. J. Clin. Endocrinol. Metab..

[43] Sones B.E., Devlin B.L. (2025). The impact of time-restricted eating on health-related quality of life: A systematic literature review. Nutr. Rev..

[44] Jefcoate P.W., Robertson M.D., Ogden J., Johnston J.D. (2023). Exploring rates of adherence and barriers to time-restricted eating. Nutrients.

[45] Robinson E., Blissett J., Higgs S. (2013). Social influences on eating: Implications for nutritional interventions. Nutr. Res. Rev..

[46] Vizthum D., Katz S.E., Pacanowski C.R. (2023). The impact of time restricted eating on appetite and disordered eating in adults: A mixed methods systematic review. Appetite.

[47] Noh J.W., Jung J.H., Park J.E., Lee J.H., Sim K.H., Park J., Kim M.H., Yoo K.-B. (2018). The relationship between age of onset and risk factors including family history and lifestyle in Korean population with type 2 diabetes mellitus. J. Phys. Ther. Sci..

